# Supplementary material for: On Learned Operator Correction in Inverse Problems
Source: SIAM J Imaging Sci. Author manuscript; Available in PMC 2024 Dec 31. (PMC7617273; doi:10.1137/20M1338460)
Supplement: Appendix [file EMS123349-supplement-Appendix.pdf]

Downloaded 05/03/21 to 5.66.2.168. Redistribution subject to CCBY license

Appendix A. An approximate model for photoacoustic tomography. Here we discuss the accurate and approximate model as previously used in [20]. In PAT a short pulse of near-infrared light is absorbed by chromophores in biological tissue. For a sufficiently short pulse, the quantity of interest will result as a spatially varying pressure increase x, which will initiate an ultrasound pulse (*photoacoustic effect*), that then propagates to the tissue surface. The measurement consists of the detected waves in space-time at the boundary of the tissue; this set of pressure time series constitutes the measured photoacoustic data y.

For the forward model, this acoustic propagation is commonly modeled by an initial value problem for the wave equation [11],

$$(\partial_{tt} - c^2\Delta)p(\mathbf{x},t) = 0, \quad p(\mathbf{x},t=0) = x(\mathbf{x}), \quad \partial_t p(\mathbf{x},t=0) = 0, \text{ with } \mathbf{x} \in \mathbb{R}^2. \tag{A.1}$$

The measurement is then modeled as a linear operator $\mathcal{M}$ acting on the pressure field $p(\mathbf{x},t)$ restricted to the boundary of the computational domain Ω and a finite time window

$$y = \mathcal{M}\, p_{|\partial\Omega\times(0,T)}. \tag{A.2}$$

Together, (A.1) and (A.2) define the linear forward model that we consider in this study:

$$Ax = y \tag{A.3}$$

© 2021 SIAM. Published by SIAM under the terms of the Creative Commons 4.0 license

from initial pressure x to the measured time series y. This accurate forward model can be simulated by a pseudospectral time-stepping model as outlined in [42, 43].

For the approximate model, we can exploit the fact that in our case the measurement points lie on a line ($\mathbf{x}_2 = 0$) outside the support of x; the pressure there can be related to x by [11, 28]

$$p(\mathbf{x}_1, t) = \frac{1}{c^2}\mathcal{F}_{k_1}\left\{\mathcal{C}_\omega\left\{B(k_1,\omega)\tilde{x}(k_1,\omega)\right\}\right\}, \tag{A.4}$$

where $\tilde{x}(k_1,\omega)$ is obtained from $\hat{x}(k)$ via the dispersion relation $(\omega/c)^2 = k_1^2 + k_2^2$ and $\hat{x}(k) = \mathcal{F}_{\mathbf{x}}\{x(\mathbf{x})\}$ is the 2-dimensional Fourier transform of $x(\mathbf{x})$. $\mathcal{C}_\omega$ is a cosine transform from ω to t, $\mathcal{F}_{k_1}$ is the 1-dimensional inverse Fourier transform from k_1 to $\mathbf{x}_1$ on the detector. The weighting factor,

$$B(k_1,\omega) = \omega / \left(\operatorname{sgn}(\omega)\sqrt{(\omega/c)^2 - k_1^2}\right), \tag{A.5}$$

contains an integrable singularity which means that if (A.4) is evaluated by discretization on a rectangular grid (and thus enabling the application of FFT for efficient calculation), then aliasing in the measured data $p(\mathbf{x}_1, t)$ results. Consequently, evaluating (A.4) using FFT leads to a *fast but approximate* forward model. In fact, we can control the degree of aliasing, by avoiding the singularity, that means in practice all components of B for which $k_1^2 > (\omega/c)^2 \sin^2\theta_{\max}$ are set to zero. This is equivalent to assuming only waves arriving at angles up to $\theta_{\max}$ from normal incidence are detected. We note, that there is a trade-off: the greater the range of angles included, the greater the aliasing. Finally, this results in a thresholded weighting factor $\widetilde{B}$ and hence the relation (A.4) using $\widetilde{B}$ defines the approximate model for this study: $\widetilde{A}x = y$.

Appendix B. Addition to theoretical results. In this section, we only investigate the question of closeness of minimizers, without investigating if the minimizers of $\mathcal{L}_\Theta$—that involves a nonlinear operator in the data term—can be identified efficiently using a gradient descent based algorithm. To answer this question, we will assume that the learned corrected operator A_Θ approximates the ground-truth operator A sufficiently well, uniformly on some manifold $\mathcal{D}$ that contains the minimizer of $\mathcal{L}$. These assumptions represent the situation of a well-fit forward approximation on the data manifold $\mathcal{D}$ that we assume all relevant reconstructions to lie on.

While it is difficult to check these assumptions in practice, the purpose of this discussion is to give a more complete theoretical view of the problem at hand, demonstrating that under sufficient assumptions closeness of forward operators is sufficient to deduce closeness of minimizers. However, this does not guarantee that the minimum can be found with a gradient descent algorithm or that a gradient descent algorithm even stays on the manifold $\mathcal{D}$ of good approximation quality. As a theoretical underpinning for the experiments conducted in this paper, Theorem 4.9 should hence instead be considered as a the main theorem.

Proposition B.1 (proximity of minimizers). *Denote by $\mathcal{D} \subset X$ the manifold of possible reconstructions that the operator approximation was trained on using empirical risk mini-*

© 2021 SIAM. Published by SIAM under the terms of the Creative Commons 4.0 license

Downloaded 05/03/21 to 5.66.2.168. Redistribution subject to CCBY license
